# A method of quantitative chemiluminescence immunoassay for the concentration of Growth differentiation factor-15

**DOI:** 10.1016/j.mex.2024.102572

**Published:** 2024-01-15

**Authors:** Ju Zhang, Jiajia Zhang, Ting Wu, Peipei Jin, Chengyi Huang

**Affiliations:** a Department of Laboratory Medicine, The First Affiliated Hospital of USTC, Division of Life Sciences and Medicine, University of Science and Technology of China, Hefei, Anhui, China; b Core Unit of National Clinical Research Center for Laboratory Medicine, Hefei, Anhui, China; c Department of Clinical Laboratory Diagnostics, Bengbu Medical College, Bengbu, Anhui, China; d Maccura Biotechnology Co., Ltd, Chengdu, Sichuan, China

**Keywords:** GDF-15, Heart failure, Inflammatory Biomarkers, Chemiluminescence Immunoassay, Cardiovascular disease, Chemiluminescence Immunoassay

## Abstract

Growth differentiation factor-15 (GDF-15), a member of the transforming growth factor (TGF-β) superfamily, and is expressed and secreted in response to inflammation, oxidative stress and hypoxia. It has been shown in several studies to be a predictor of heart failure. However, the only kits available on the market are ELISA kits, which are costly and error-prone and are not conducive for clinical use. Here, we developed a chemiluminescence kit which optimized the reaction conditions and the reaction time was reduced to 10 min. We further proved that it can be used to measure GDF-15 in serum or plasma accurately and fastly, and provide additional information for the diagnosis of heart failure disease. Methodological comparison and clinical study verified this method is a reliable, economical and highly automated blood test method.

• All necessary steps and the reagents needed are provided.
• Reliability of the chemiluminescence immunoassay was verified by analyzing serum GDF-15 levels from different groups.
• GDF-15 can provide clinicians with reliable prediction and disease assessment of heart failure.

## Graphical abstract

**Figure:**
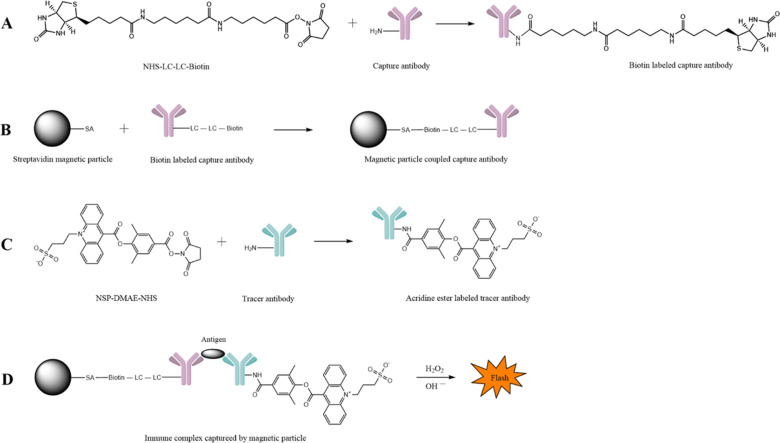

Specifications table

**Table:** 

Subject area:	Immunology and Microbiology
More specific subject area:	Clinical laboratory diagnostics
Name of your method:	Chemiluminescence Immunoassay
Name and reference of original method:	ELISA / J. Yin, Z. Zhu, D. Guo, A. Wang, N. Zeng, X. Zheng, Y. Peng, C. Zhong, G. Wang, Y. Zhou, C.S. Chen, J. Chen, Y. Zhang, J. He, Increased Growth Differentiation Factor 15 Is Associated with Unfavorable Clinical Outcomes of Acute Ischemic Stroke, Clin Chem 65(4) (2019) 569–578. doi: 10.1373/clinchem.2018.297879.
Resource availability:	N.A.

## Method details

### Introduction

Due to the population aging, the prevalence and fatality rates of cardiovascular diseases are on the rise. Heart failure, which is the primary cause of death related to cardiovascular diseases, is not an isolated ailment but rather a shared pathway for various heart conditions. Currently, NT-proBNP is the widely employed prognostic biomarker for heart failure (HF). However, NT-proBNP does not encompass the complete pathophysiology of HF, and there may be other biomarkers that can reflect different disease mechanisms [1].

**Fig. 1:**
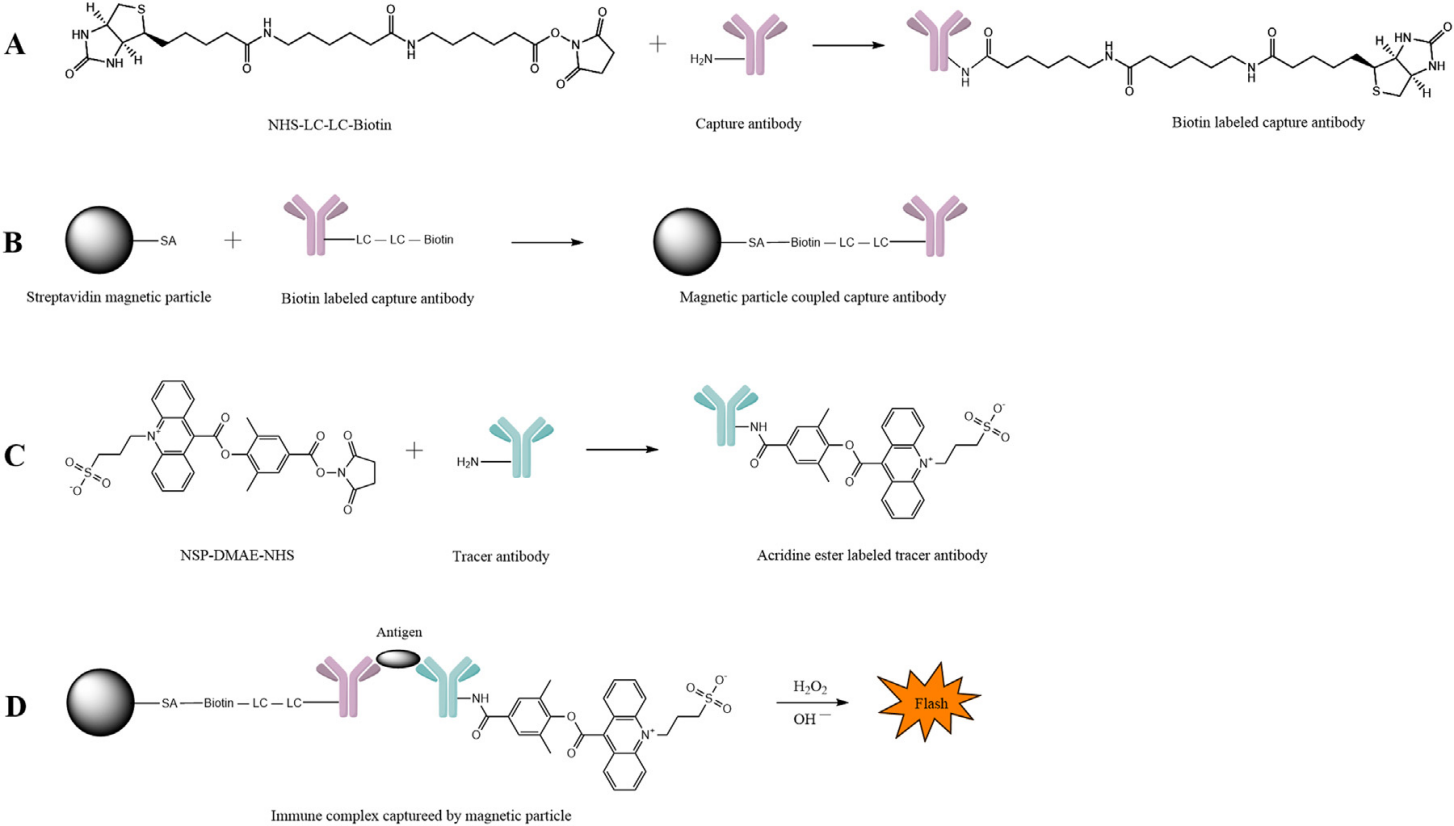
The reaction principle of quantitative chemiluminescence immunoassay for concentration determination of GDF-15. A. Mechanism illustration of the biotin labeling to capture antibody. The *N*-Hydroxysuccinimide on NHS-LC-LC-Biotin reacts with the primary amino group of the capture antibody to form an amide bond. B. The capture antibody is coupled to the magnetic particle by a specific reaction of biotin and streptavidin. C. The principle of NSP-DMAE-NHS coupling to tracer antibody. D. Detection principle of the chemiluminescence immunoassay. The antigen concentration is proportional to the relative light unit (RLU).

GDF-15, a member of the transforming growth factor (TGF-β) superfamily, is typically expressed in low concentrations across various organs [2]. However, in diseases affecting the heart [3], kidney [4], lung [5], and liver [6], its expression is elevated. As a result, GDF-15 can serve as a potential predictor for systemic processes in heart failure. Currently, only ELISA kits for scientific research are available in the market [7]. These kits are complex, prone to errors, and exhibit low sensitivity. To address these limitations, we have developed a chemiluminescence kit based on streptavidin superparamagnetic particles. This kit offers a larger specific surface area, allowing for more antibody coating. It is also more sensitive and can be easily automated, thereby compensating for the drawbacks of ELISA kits (Fig. 1).

## Method details

### Materials

#### Reagents

• Streptavidin magnetic particles (10 mg/mL)
• NHS-LC-LC-Biotin (10 mM in DMSO or DMF, Preparation before use)
• 1 mol/L Tris
• Phosphate-buffered saline (PBS)
• Storage buffer 1 (0.1 % Tween20, 0.5 % PC-950, 1 % bovine serum albumin (BSA) in PBS)
• NSP-DMAE-NHS (10 mM in DMSO or DMF)
• 10 mg/mL l-Lysine
• Desalting Columns
• Blocking reagent (Vitamin H in DMSO or DMF)
• Storage buffer 2 (0.01 % TX-100, 0.3 % PC-300, 1 % Casein in PBS)
• GDF15-specific monoclonal antibody (Capture antibody and tracer antibody)
• Pre-excitation fluid :H_2_O_2_
• Excitation fluid: NaOH
• Acridine ester

#### Procedure

(1) Biotin labeling reaction
  a) The capture antibody was dialyzed using PBS and diluted with PBS to 1mg/ml, 1 mg (1 mL) of the capture antibody after dialysis was taken into the sample tube, and added 3.3 µL 10 mM NHS-LC-LC-Biotin. Incubate the mixture for 30 min at room temperature with stirring.
  b) After the reaction was completed, 10 µl Tris buffer (1 mol/L) was added, mixed and reacted at room temperature for 10 min.
  c) Remove the non-reacted and hydrolyzed biotin reagent by desalting Columns.
(2) Magnetic particle coupling
  a) Add 2 mL of streptavidin magnetic particles to a normal 15 mL Flacon tube. Make sure all magnetic particles are added to the tube.
  b) Add 8 mL storage buffer 1 to vial and mix, place it on the magnetic separator, wait for the liquid to clear, discard the supernatant, repeat the above cleaning step three times, then resuspend with 8 ml storage buffer 1.
  c) Add 40 µL biotinylated capture antibody, mix well, and react on a rocker for 0.5 h at room temperature.
  d) Repeat buffer wash in step 2(b).
  e) Add 200 µL blocking reagent to vial and incubate at room temperature on a rocker for 15 min.
  f) Repeat buffer wash in step 2(b).
  g) Add 42 mL storage buffer 1 to dilute the magnetic particles concentration to 0.4 mg/ml.
(3) Acridine ester labeling reaction
  a) The tracer antibody was dialyzed using PBS and diluted with PBS to 1mg/ml, 1 mg (1 mL) of the tracer antibody after dialysis was taken into the sample tube, add 10 µL NSP-DMAE-NHS (10 mM) to sample tube. React and protected from light on a rocker for 1 h at room temperature.
  b) Add 20 µL l-lysine (10 mg/mL), and mix immediately, and react at room temperature on a blood mixing apparatus for 10 min under the condition of avoiding light.
  c) The finished tracer antibodies need to be purified using a desalting column to remove excess acridine ester according to manufacturer's instructions.
  d) Acridine ester labeled tracer antibodies were diluted to 0.1 µg/mL using Storage buffer 2.
(4) Reaction process
  a) The i 3000 Automatic Chemiluminescence Analyzer from Maccura Biotechnology Co., Ltd was used for detection. 10 µl sample, 50 µl capture reagent, and 100 µl tracer reagent were added to reaction vials and mixed, After 10 min reacting at 37 ℃, 200 µL pre-excitation fluid (H_2_O_2_) and 200 µL excitation fluid (NaOH) were then added and measured the relative light unit (RLU). Because acridine ester luminescence belongs to the flash type, so our light signal acquisition time is only 100 ms. The concentration of GDF-15 in the sample is proportional to RLU.

## Method validation

To analyze the effectiveness of this chemiluminescence immunoassay, we performed methodological comparisons with commercially available ELISA kits (Human GDF-15 Quantikine QuicKit ELISA, Catalog: QK957, R&D systems). A total of 254 samples were used including children, the elderly, pregnant women, dialysis patients and heart patients. We also analyzed serum GDF-15 levels in apparently healthy controls, patients with metabolic syndrome, acute coronary syndrome, atrial fibrillation and chronic heart failure. Mann Whitney test was used for comparison between groups. Spearman's rank correlation was used for correlation analysis.

**Fig. 2:**
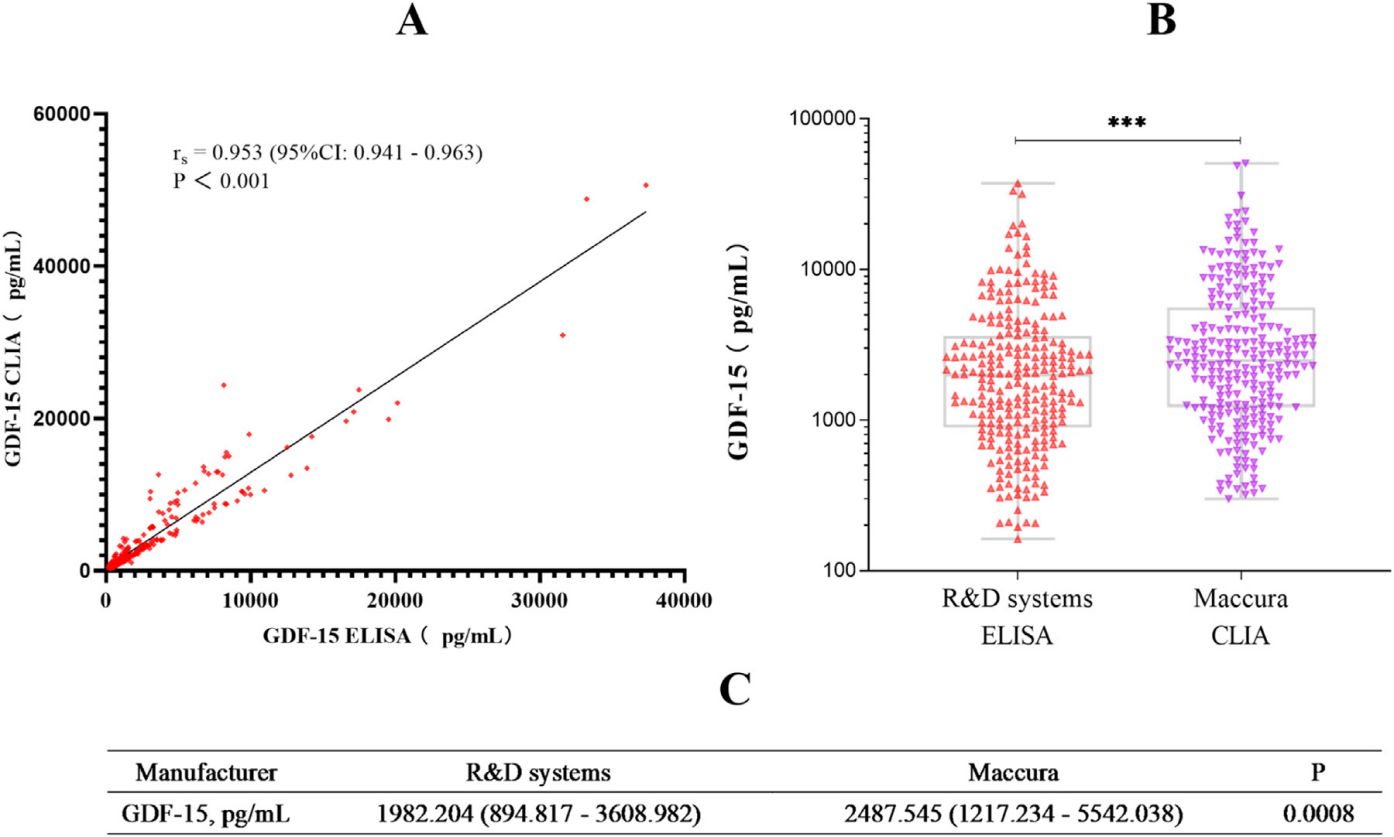
A, Correlation between GDF-15 levels in 254 serum samples were measured by CLIA vs. ELISA. Correlation coefficient and P-value were calculated using the Spearman's rank correlation. B, Differences in GDF-15 concentrations between CLIA and ELISA. C, Median and interquartile range (IQR) of GDF-15 concentrations for CLIA and ELISA.

**Fig. 3:**
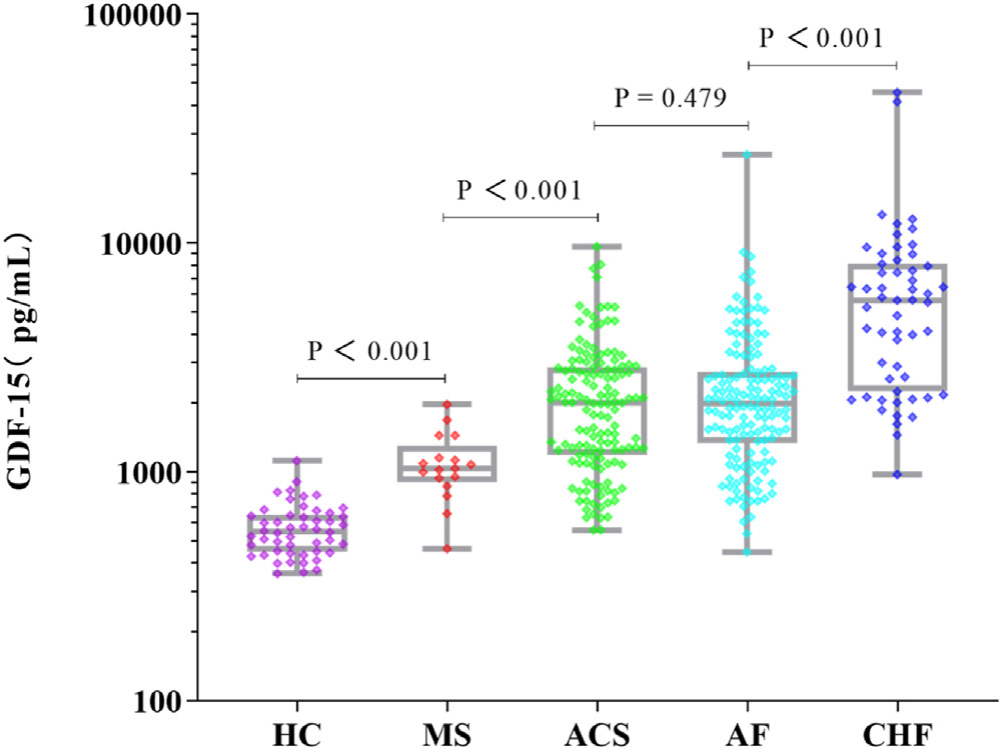
Reliability of our chemiluminescence immunoassay was tested by analyzing serum GDF-15 levels from HC (healthy control), MS (metabolic syndrome), ACS (acute coronary syndrome), AF (atrial fibrillation), CHF (chronic heart failure) groups. Statistical analysis of blood serum data was conducted using Mann Whitney test.

Fig. 2 shows a comparison of our established chemiluminescence immunoassay with a commercially ELISA kit. Spearman's rank correlation was 0.953 (95 %CI: 0.941–0.963), indicating a significant correlation between the two assay (*P* < 0.001). Although the median values of CLIA and ELISA were significantly different, and the GDF-15 concentration of CLIA was significantly higher than that of ELISA. There were significant differences in GDF-15 concentration among different populations. GDF-15 levels were significantly elevated in patients with metabolic syndrome, acute coronary syndrome, atrial fibrillation and chronic heart failure (Fig. 3). These data are consistent with previous findings that GDF-15 levels raise significantly in cardiovascular disease compared to healthy controls [1,[8], [9], [10]]. Therefore, we conclude that our chemiluminescence immunoassay is a reliable method for analyzing serum GDF-15 concentration in clinical and in vivo studies.

## Ethics statements

The study has been granted an exemption from requiring ethics approval by the Institutional Review Boards and ethics committee at University of science and technology of China as well as the First Affiliated Hospital of USTC, and written informed consent was not required for these reasons:

1. This research using medical records and biological specimens obtained in previous clinical care
2. The risk to the subject of the study is not greater than the minimal risk.
3. The exemption from informed consent will not adversely affect the rights and health of the subject.
4. Subjects' privacy and personally identifiable information is protected.

## CRediT authorship contribution statement

**Ju Zhang:** Methodology, Investigation. **Jiajia Zhang:** Data curation, Investigation. **Ting Wu:** Validation, Data curation. **Peipei Jin:** Conceptualization, Writing – review & editing. **Chengyi Huang:** Validation, Writing – original draft.

## Declaration of competing interest

The authors declare that they have no known competing financial interests or personal relationships that could have appeared to influence the work reported in this paper.

## Data Availability

Data will be made available on request.
